# Few-Shot Adaptation of Foundation Vision Models for PCB Defect Inspection

**DOI:** 10.3390/jimaging11110415

**Published:** 2025-11-17

**Authors:** Sang-Jeong Lee

**Affiliations:** Multimodal AX Business Team, LG CNS Co., Ltd., Seoul 07795, Republic of Korea; sjlee89@lgcns.com

**Keywords:** automated optical inspection (AOI), few-shot learning, Low-Rank Adaptation (LoRA), parameter-efficient tuning (PEFT), printed circuit board (PCB), Visual Prompt Tuning (VPT)

## Abstract

Automated Optical Inspection (AOI) of Printed Circuit Boards (PCBs) suffers from scarce labeled data and frequent domain shifts caused by variations in camera optics, illumination, and product design. These limitations hinder the development of accurate and reliable deep-learning models in manufacturing settings. To address this challenge, this study systematically benchmarks three Parameter-Efficient Fine-Tuning (PEFT) strategies—Linear Probe, Low-Rank Adaptation (LoRA), and Visual Prompt Tuning (VPT)—applied to two representative foundation vision models: the Contrastive Language–Image Pretraining Vision Transformer (CLIP-ViT-B/16) and the Self-Distillation with No Labels Vision Transformer (DINOv2-S/14). The models are evaluated on six-class PCB defect classification tasks under few-shot (k = 5, 10, 20) and full-data regimes, analyzing both performance and reliability. Experiments show that VPT achieves 0.99 ± 0.01 accuracy and 0.998 ± 0.001 macro–Area Under the Precision–Recall Curve (macro-AUPRC), reducing classification error by approximately 65% compared with Linear and LoRA while tuning fewer than 1.5% of backbone parameters. Reliability, assessed by the stability of precision–recall behavior across different decision thresholds, improved as the number of labeled samples increased. Furthermore, class-wise and few-shot analyses revealed that VPT adapts more effectively to rare defect types such as Spur and Spurious Copper while maintaining near-ceiling performance on simpler categories (Short, Pinhole). These findings collectively demonstrate that prompt-based adaptation offers a quantitatively favorable trade-off between accuracy, efficiency, and reliability. Practically, this positions VPT as a scalable strategy for factory-level AOI, enabling the rapid deployment of robust defect inspection models even when labeled data is scarce.

## 1. Introduction

Printed Circuit Boards (PCBs) are a fundamental component in modern electronics manufacturing, and their reliable inspection is essential for ensuring product quality and reducing downstream failures [1]. Automated Optical Inspection (AOI) systems are widely used to detect various defect types—including open circuits, shorts, spurious copper, and pinholes—but developing accurate machine-learning models for such visual defect recognition remains challenging. A major limitation is the scarcity of annotated data, since labeling PCB defects requires expert knowledge and significant manual effort [2]. These limitations hinder the development of accurate and reliable deep-learning models. In this context, ‘accurate’ refers to high predictive correctness (measured by metrics such as Accuracy and Macro-F1), while ‘reliable’ refers to the model’s ability to provide well-calibrated confidence scores that reflect the true likelihood of correctness, quantified by threshold-robust metrics like the macro–Area Under the Precision–Recall Curve (macro-AUPRC). This constraint motivates the study of label-efficient learning approaches that can achieve high performance with limited supervision.

Traditional PCB inspection algorithms have largely relied on Convolutional Neural Networks (CNNs) trained in a fully supervised manner (e.g., ResNet) [3]. While achieving high performance when sufficient labeled data are available, CNN-based approaches tend to degrade under low-label conditions. In addition, detection-oriented pipelines, though capable of defect localization, require bounding-box annotations and post-processing to localize each defect.

Recently, the advent of large-scale foundation vision models such as the Vision Transformer (ViT) [4] and multimodal extensions like Contrastive Language–Image Pretraining (CLIP) [5], and Self-Distillation with No Labels Vision Transformer (DINO) [6] has reshaped computer vision research. A foundation model refers to a neural network pretrained on broad and diverse datasets that can be efficiently adapted to downstream tasks through limited fine-tuning. These models offer transferable representations; however, the optimal adaptation strategy for industrial and data-limited settings—such as PCB inspection—remains insufficiently explored.

To address this gap, we investigate three Parameter-Efficient Fine-Tuning (PEFT) techniques: Linear Probing, Visual Prompt Tuning (VPT) [7], and Low-Rank Adaptation (LoRA) [8]. In contrast to full fine-tuning, which updates all parameters of a pretrained model, PEFT methods modify only a small fraction (typically < 2%), thereby reducing computational cost and overfitting risk. The few-shot setting, in which only k-labeled examples per class are available, serves as a realistic benchmark for evaluating such approaches [9]. Despite promising results in natural image and language benchmarks, their performance on manufacturing data—especially PCB defect imagery—has not been systematically assessed.

Beyond industrial contexts, parameter-efficient fine-tuning (PEFT) strategies have shown high performance across various non-manufacturing domains. In medical imaging, PEFT has enabled adaptation of foundation models such as Vision Transformers and CLIP to limited annotated data in histopathology and radiography tasks, improving diagnostic accuracy and calibration with less than 2% trainable parameters [10,11]. In anomaly detection, LoRA and prompt-based adaptation have been successfully applied to few-shot defect segmentation and out-of-distribution detection, achieving comparable accuracy while maintaining low computational overhead [12]. These studies collectively demonstrate that PEFT methods generalize effectively across diverse visual domains, supporting their potential applicability to industrial inspection tasks such as PCB defect classification.

In industrial inspection, performance evaluation must extend beyond simple accuracy. Reliability describes the alignment between predicted probabilities reflect actual correctness and is typically quantified using metrics such as threshold-robust precision–recall behavior. A comprehensive evaluation should therefore consider accuracy, class-wise balance, calibration, and efficiency simultaneously to determine the most practical method for deployment.

To address this challenge, this study conducts a systematic benchmark by quantitatively comparing three distinct PEFT strategies (Linear Probe, LoRA, and VPT) applied to two representative foundation models. The comparison is performed across multiple data regimes (few-shot k = 5, 10, 20, and full data) using a standardized PCB dataset and a comprehensive set of evaluation metrics. Also, we define few-shot adaptation as the process of fine-tuning a foundation model for a specific downstream task using a very limited number of labeled examples, such as k = 5, 10, or 20 samples per class.

Our contributions are summarized as follows:

1. We construct a PCB defect classification benchmark derived from aligned DeepPCB [13] image pairs, enabling controlled evaluation of foundation model adaptation under limited-label conditions.
2. We provide a comparative analysis of Linear Probe, LoRA, and VPT, quantifying trade-offs in accuracy, parameter efficiency, and reliability. Linear Probe serves as the conventional baseline, updating only the classification head on frozen backbones and representing minimal adaptation cost. LoRA introduces small low-rank matrices within transformer layers, efficiently updating internal representations. VPT injects learnable prompt tokens at the input level, offering a complementary mechanism that modifies attention without altering pretrained weights.
3. We demonstrate that parameter-efficient tuning consistently improves few-shot classification accuracy and calibration compared with linear probing, offering practical insights for adopting foundation models in manufacturing quality assurance.

Accordingly, the aim of this paper is to quantitatively compare these three PEFT paradigms across both full-data and few-shot settings using the DeepPCB dataset, examining not only accuracy but also calibration-based reliability and deployment feasibility. This work bridges the gap between general-purpose vision foundation models and industrial inspection requirements, establishing a reference framework for future research on detection-level and reliability-aware PEFT adaptation in PCB AOI systems.

The remainder of this paper is organized as follows. Section 2 reviews related works on PCB inspection, foundation vision models, and parameter-efficient fine-tuning strategies. Section 3 describes the dataset, backbone architectures, and parameter-efficient tuning configurations used in this study. Section 4 presents quantitative and qualitative results under few-shot and full-data settings, while Section 5 discusses the implications for reliability, label efficiency, and deployment. Finally, Section 6 concludes the paper with a summary of the main findings and outlines potential directions for future research.

## 2. Related Works

### 2.1. PCB Defect Inspection

Early studies on PCB defect inspection primarily relied on classical image processing and template-matching techniques, comparing a tested PCB against a golden reference to identify deviations [14]. While suitable for large-scale (coarse) defects, such methods were sensitive to illumination, alignment, and noise, which limited their robustness in production environments. With the advent of deep learning, CNNs improved defect detection and classification performance. Residual learning architectures such as ResNet became common baselines for PCB inspection by enabling deeper feature extraction [3]. Datasets such as DeepPCB [13] facilitated benchmarking of these approaches, yet fully supervised CNNs still depend on extensive annotation and often exhibit degraded performance when domain conditions (camera, lighting, or board design) shift. Recently, several studies have explored more advanced deep learning approaches for PCB and industrial defect detection. Zhao et al. proposed an Automated Defect Identification System in Printed Circuit Boards using region-based convolutional neural networks, achieving accurate localization and classification of fine defects under real manufacturing conditions [15]. In parallel, Soni et al. presented a comprehensive survey on Deep Learning for Automatic Vision-Based Recognition of Industrial Surface Defects, highlighting the growing importance of data-efficient and transferable models for industrial visual inspection [16]. These recent works further underscore the ongoing shift toward deep learning-driven, scalable inspection frameworks in manufacturing. These limitations motivated the exploration of transferable and label-efficient learning paradigms suitable for data-constrained industrial settings.

### 2.2. Foundation Vision Models

The emergence of large-scale pretrained vision models has transformed visual recognition. The ViT showed that self-attention mechanisms can match or exceed CNNs in image understanding tasks [4], while the CLIP model enabled zero-shot transfer through large-scale image–text alignment [5]. Self-supervised encoders such as DINOv2 have further improved feature robustness at scale [6]. These advances suggest that foundation models provide generalized and transferable representations that could benefit industrial inspection tasks with limited labeled data. However, their direct adaptation to manufacturing domains, including PCB-AOI, remains insufficiently explored, particularly in terms of label efficiency and calibration reliability.

### 2.3. Parameter-Efficient Tuning Strategies

Full fine-tuning of foundation models is computationally expensive and can easily overfit in few-shot regimes [7]. PEFT methods address this issue by updating only a small subset of model parameters. Linear Probing freezes the backbone and trains only the final classification layer, providing a minimal-cost baseline [4]. VPT prepends learnable prompt tokens to the patch embeddings, guiding attention toward task-relevant regions without modifying pretrained weights [7]. LoRA inserts small low-rank matrices within transformer attention and layers, achieving performance comparable to full fine-tuning with a fraction of trainable parameters [8]. While these strategies have shown promise for natural images and other visual domains such as medical imaging and remote sensing [8,9,10,11,12], there has been no systematic comparison of their behavior in manufacturing-oriented contexts—particularly when both data efficiency and reliability are required.

### 2.4. Reliability and Calibration

Beyond accuracy, reliability—the alignment between model confidence and correctness—is essential in industrial inspection, where false negatives can lead to costly downstream errors. Modern neural networks are often miscalibrated, requiring explicit evaluation using calibration metrics such as Expected Calibration Error (ECE) [17]. In addition, selective prediction frameworks such as risk–coverage analysis [18] and cost-sensitive evaluation [19] offer complementary ways to assess deployment reliability by explicitly modeling the trade-off between risk and coverage or between error and cost. However, such analyses remain rare in PCB-AOI research, which typically emphasizes accuracy alone. This gap highlights the need for comprehensive evaluation frameworks that jointly assess both predictive performance and reliability when adapting foundation vision models to manufacturing inspection tasks.

## 3. Materials and Methods

### 3.1. Dataset and Preprocessing

We used the DeepPCB dataset [13], a publicly available benchmark for printed circuit board (PCB) defect detection and classification. DeepPCB was selected because it provides consistent annotations, aligned image pairs, and multi-class labeling consistent with automated optical inspection (AOI) practices. The dataset contains 1500 image pairs (template–tested), each with a size of 640 × 640 pixels, including six annotated defect categories: mousebite, open, pinhole, short, spur, and spurious copper. Each annotation provides bounding box coordinates in pixel space, representing defective regions suitable for both detection and classification tasks. DeepPCB was chosen as the benchmark dataset in this study because it offers annotation consistency, geometric registration between reference and tested images, and coverage of major PCB defect types. These characteristics make it the most reproducible and widely adopted dataset for benchmarking PCB automated optical inspection (AOI) models. Furthermore, the dataset size and diversity are sufficient to support both few-shot and full-data experiments, ensuring fair evaluation of data-efficient learning strategies.

**Figure 1:**
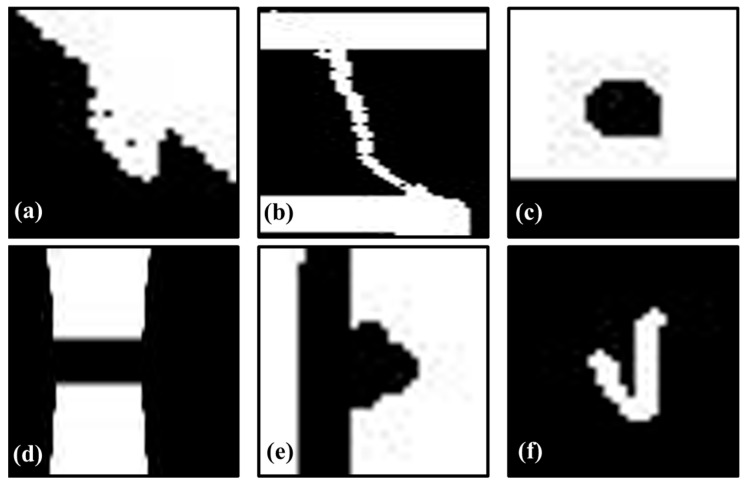
Example of defect patch extraction from DeepPCB dataset: (**a**) Mousebite, (**b**) Open, (**c**) Pinhole, (**d**) Short, (**e**) Spur, and (**f**) Spurious copper.

To reformulate the task as defect classification, each annotated bounding-box region was cropped to generate individual defect patches (Figure 1). To clarify, we used the bounding box annotations provided with the dataset to extract defect patches directly from the ‘tested’ images. We did not use the ‘template’ images or create difference images for this classification task, as our goal was to classify the appearance of the defect itself. Crops were resized according to the backbone input requirements—224 × 224 pixels for CLIP-ViT-B/16 [5] and 518 × 518 pixels for DINOv2-S/14 [6]—and normalized to the [0, 1] range. To improve generalization, we applied random horizontal and vertical flips (probability = 0.5) and mild brightness (±10%) and contrast (±5%) adjustments. Stronger augmentations such as rotation or color jitter were deliberately avoided to preserve the integrity of the few-shot setting and prevent label confusion. The dataset was divided into training, validation, and test splits with a 7:1:2 ratio, stratified by defect class to preserve class composition. This result was summarized in Table 1.

For few-shot experiments, we subsampled the training split to retain only k ∈ {5, 10, 20} labeled samples per class, keeping the validation and test splits fixed. All random sampling was performed using a fixed seed (42) to ensure reproducibility. During quality control, approximately 5% of the image pairs exhibiting slight misalignment between the reference and tested boards were manually corrected.

### 3.2. Backbone Architectures

We selected two representative vision foundation models that embody distinct pretraining paradigms and complementary inductive biases (Table 2).

CLIP-ViT-B/16 is a 12-layer Vision Transformer with a patch size of 16 × 16 and a 768-dimensional embedding space, pretrained on approximately 400 million image–text pairs via contrastive learning [5]. The CLIP backbone represents a multimodal supervision paradigm, capturing semantic and globally contextual features that intended for generalization across diverse domains. This property is expected to benefit defect recognition tasks that require semantic differentiation among visually similar PCB regions.

DINOv2-S/14 is a self-supervised Vision Transformer comprising 12 layers with a 14 × 14 patch size and a native resolution of 518 × 518 pixels, trained on 142 million unlabeled natural images [6]. Unlike CLIP, DINOv2 learns representations through self-distillation without labels, resulting in representations sensitive to texture and structure.

This inductive bias is particularly relevant for PCB inspection, where local, fine-grained visual patterns such as copper edges or micro defects play an important role. CLIP and DINOv2 together provide complementary perspectives—semantic versus structural representation—allowing a balanced evaluation of adaptation strategies for industrial imagery. Both backbones were implemented using the timm library (v0.9.16) and initialized with publicly available pretrained weights.

### 3.3. Parameter-Efficient Tuning 

**Figure 2:**
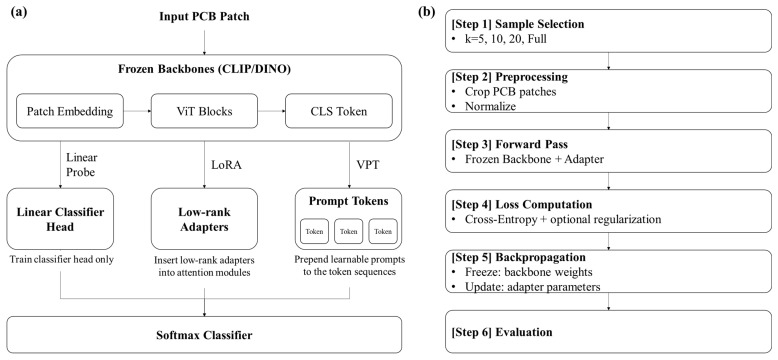
Mechanistic overview and training flow of parameter-efficient tuning (PEFT) for foundation vision models. (**a**) Architecture comparison showing where each adapter (Linear, LoRA, VPT) modifies the frozen backbone. (**b**) End-to-end few-shot training flow highlighting.

Figure 2 illustrates the overall PEFT adaptation pipeline. We selected these three PEFT techniques (Linear Probe, LoRA, and VPT) as they represent three distinct and widely adopted paradigms for parameter-efficient model adaptation. Linear Probe serves as the minimal baseline, updating only the final classification head while the backbone remains frozen. LoRA represents ‘adapter-based’ methods, which inject small, trainable low-rank matrices into the backbone’s layers to efficiently update internal representations. VPT represents prompt-based methods, which add learnable tokens to the input sequence to guide the model’s attention without altering pretrained backbone weights. We compared three parameter-efficient tuning (PEFT) approaches:

- Linear Probe: The backbone encoder was frozen, and only the final classification head (a linear layer) was trained. This strategy serves as a baseline for transfer learning efficiency. Let $f_{\theta }(x)\in R^{d}$ be the frozen backbone embedding. Linear learns only $W\in R^{C\times d}$, $b\in R^{C}$ and predicts ${\hat{y}=softmax(Wf}_{\theta }\left(x\right)+b)$. Trainable params are limited to the classifier head.
  where
  ▪ x: input PCB patch image;
  ▪ $f_{\theta }(x)$: feature vector from the frozen backbone;
  ▪ *d*: feature dimension (e.g., 768 for ViT-B/16);
  ▪ *C*: number of defect classes (6 in DeepPCB);
  ▪ *W*: linear classifier weight mapping features to logits;
  ▪ *b*: bias term per class;
  ▪ $\hat{y}$: class probability vector after softmax.
  Only *W*, *b* are trained; all backbone weights *θ* remain frozen.
- Low-Rank Adaptation (LoRA): Trainable low-rank matrices were inserted into the query, key, value, projection, and MLP linear layers of the transformer. In LoRA, rank (*r*) defines the dimensionality of the low-rank update subspace, α controls the scaling of the injected adaptation, and dropout (0.05) regularizes the adapter to prevent overfitting. These settings (*r* = 8, *α* = 16) follow established practice and yield an adaptation module with a low parameter count (*r* = 8). Original backbone weights were frozen, and only LoRA adapters plus the classifier head were updated. For each target linear map $W_{0}\in R^{m\times n}\;\left(e.g.,\;W_{q},W_{k},W_{v},W_{o}\;and\;MLP\;layers\right)$, LoRA learns a low-rank update:
  (1) $${W=W}_{0}+∆W,\;∆W=BA,\;A\in R^{r\times n},\;B\in R^{m\times r}$$
  where
  ▪ $W_{0}$: frozen pretrained weight matrix of the backbone;
  ▪ Δ*W*: trainable low-rank residual added to $W_{0}$;
  ▪ *A*: projection matrix from input to low-rank latent space;
  ▪ *B*: reconstruction matrix back to output space;
  ▪ *r*: rank of the update (e.g., 8), controlling capacity;
  ▪ *m*, *n*: dimensions of the linear layer in transformer;
  ▪ α: scaling factor applied to *ΔW* (effective learning rate multiplier).

LoRA keeps $W_{0}$ frozen and trains only *A*, *B*. This reduces the parameter count by more than 98%.

r controls capacity; α scales ΔW (effective step *α*/*r*); dropout (0.05) regularizes adapters. Backbone weights $W_{0}$ are frozen; only (A,B) and the classifier are trained.

- Visual Prompt Tuning (VPT): Learnable prompt tokens were prepended to the input sequence at every transformer layer (“deep prompting”), with a prompt length of 8 tokens. The backbone remained frozen. Prompts were initialized randomly and optimized during training. For a ViT with patch tokens $X=\left[x_{cls,}x_{1},\ldots ,x_{N}\right]$, VPT prepends learnable prompt tokens $P_{l}\in R^{L\times d}$ at every layer l:
  (2) $$Z_{l}=\left[x_{cls,}P_{l},\;X\right]\;\vec{MSA/MLP}\;Z_{l+1}$$
  where
  ▪ $x_{cls}$: classification token representing global information;
  ▪ *X*: sequence of patch embeddings from the PCB image;
  ▪ *N*: number of image patches;
  ▪ $P_{l}$: prompt token set for layer ℓ (learnable parameters);
  ▪ *L*: number of prompt tokens per layer (e.g., 8);
  ▪ *d*: embedding dimension;
  ▪ $Z_{l}$: concatenated token sequence entering layer *l*.

Prompts act as soft attention anchors, biasing self-attention to defect-relevant structures while leaving *θ* intact. Only $\left\{P_{l}\right\}$ and the classifier are trained.

- Complexity & intuition: Linear minimizes updates (fast but limited capacity); LoRA injects structured low-rank changes into attention/MLP subspaces; VPT performs layer-pervasive input conditioning, which is effective when cues are small/irregular (PCB traces) and label-scarce.

The three strategies differed in the number of trainable parameters: 4614, 2310, and 5382 for Linear Probe, LoRA, and VPT, respectively, corresponding to less than 1.5% of the full backbone in all cases.

### 3.4. Training Configuration

All experiments were conducted using PyTorch 2.1.0 with CUDA 12.1 on a workstation equipped with an NVIDIA RTX A6000 GPU (48 GB VRAM). Training was performed for 30 epochs under the following common setup:

- Optimizer: AdamW with β_1_ = 0.9, β_2_ = 0.999;
- Learning rate: 3 × 10^−4^, with cosine decay and no warmup;
- Batch size: 16 for both backbones;
- Weight decay: 0.05;
- Loss function: Cross-entropy loss.

We employed early model selection based on the highest validation accuracy, and the corresponding checkpoint was evaluated on the test set. Each experiment was repeated three times with different random seeds, and mean values are reported.

### 3.5. Evaluation Metrics

To comprehensively assess performance, we considered both accuracy and reliability. Since the dataset is not perfectly class-balanced (as summarized in Table 1), we report macro-averaged metrics (macro-F1 and macro-AUPRC) throughout all experiments to mitigate the influence of class frequency and provide a comparison across defect categories.

- Accuracy (Acc): The fraction of correctly classified test samples.
  (3) $$Acc=\frac{1}{N}\sum_{i=1}^{N}\left[{\hat{y}}_{1}=y_{1}\right]$$
  where *N* is the total number of test samples, $y_{1}$ is the ground-truth label, and ${\hat{y}}_{1}$ is the predicted label.
- Macro-F1: Harmonic mean of precision and recall averaged equally across classes, reducing bias toward majority classes.
  (4) $$Macro-F1=\frac{1}{C}\sum_{c=1}^{C}\frac{{2\times Precision}_{c}\times {Recall}_{c}}{{Precision}_{c}+{Recall}_{c}}$$
  With
  $${Precision}_{c}=\frac{{TP}_{c}}{{TP}_{c}+{FP}_{c}},\;{Recall}_{c}=\frac{{TP}_{c}}{{TP}_{c}+{FN}_{c}}$$
  where C is the number of classes and ${TP}_{c}$, ${FP}_{c}$, and ${FN}_{c}$ are the true-positive, false-positive, and false-negative counts for class c, respectively.
- Macro-AUPRC: Area under the precision–recall curve averaged across classes, reflecting reliability across imbalanced classes because it averages class-wise precision–recall trade-offs equally.
  (5) $$Macro-AUPRC=\frac{1}{C}\sum_{c=1}^{C}\int_{0}^{1}P_{c}(R)dR$$
  where Pc(R) denotes the precision–recall curve for class c, and the integral represents the area under that curve (AUPRC).
- Class-wise accuracy: Accuracy computed individually for each defect type to highlight class-dependent behaviors.

In addition, for few-shot experiments (k = 5, 10, 20), we compared accuracy with k-shot curves to quantify label-efficiency.

We additionally conducted experiments with two conventional CNN-based baselines (ResNet50 [2] and EfficientNet-B0 [20]) under identical training conditions to enable fair comparison. These models serve as representative baselines for assessing the relative efficiency and performance of PEFT-based approaches.

## 4. Results

### 4.1. Overall Performance in the Full-Data Training

Beyond the raw accuracies in Table 3, the relative improvement becomes clearer when comparing the error rates across all models, including the CNN baselines. ResNet50 and EfficientNet-B0, as conventional convolutional backbones, achieved approximately 99.6% accuracy under full-data training, confirming their high performance but also their dependency on large parameter counts. By contrast, the PEFT-based methods—Linear, LoRA, and VPT—achieved comparable or even superior results with less than 1.5% of trainable parameters, demonstrating significantly higher efficiency.

On CLIP-ViT-B/16, VPT reduced the error rate from 2.8% to 0.9% versus Linear (a ~68% drop) and 2.9% to 0.9% versus LoRA (a ~69% drop). On DINOv2-S/14, VPT cut errors from 3.4% to 1.2% against Linear (~65% reduction) and 3.2% to 1.2% against LoRA (~63%). Thus, even when accuracy differences are ~1.9–2.2 points, the actual number of misclassifications decreases by approximately two-thirds with VPT.

VPT achieved higher Macro-AUPRC values, showing margins of +0.006–0.008 over the next best method on both backbones (Table 3). Because AUPRC is near its ceiling, these small absolute gains indicate better precision–recall calibration across thresholds. LoRA sometimes slightly exceeded Linear on AUPRC, but these ranking differences did not yield comparable accuracy changes, suggesting less threshold separation than VPT.

Validation–test scores were similar overall, suggesting consistent generalization. On CLIP, all methods were closely matched between splits. On DINO, VPT showed a 0.7-point drop from validation to test and retained the highest observed scores among methods. The relative gains differed across backbones: with DINOv2-S/14, whose representations emphasize texture and edges, VPT reduced errors most for structurally complex defects (see class-wise results). With CLIP-ViT-B/16, several easy classes saturated early, and VPT converged earlier while achieving the highest measured accuracy at the test endpoint.

In summary, the model ranking (VPT > Linear ≈ LoRA > CNN baselines) was consistent across both foundation backbones. The character of the gains aligned with each model’s inductive bias: for DINOv2-S/14, which emphasizes textural and edge representations, VPT achieved notable large reductions in error rate, improving structurally complex defect recognition. On CLIP-ViT-B/16, several easier classes saturated early, and VPT converged faster and reached higher performance at the test endpoint.

### 4.2. Few-Shot Ablations and Label Efficiency

**Figure 3:**
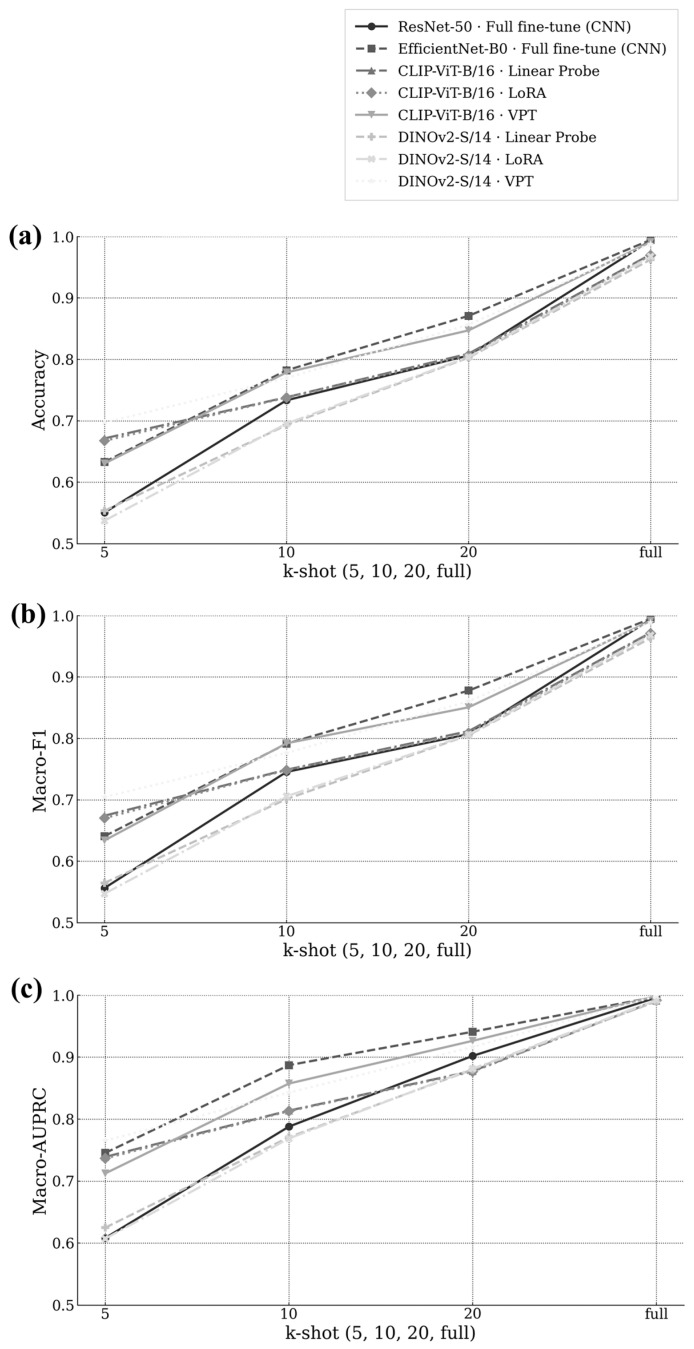
Few-shot performance curves (k = 5 to k = full) comparing baselines with Linear, LoRA, and VPT across (**a**) accuracy, (**b**) macro-F1, and (**c**) macro-AUPRC.

Figure 3 plots k = 5 to k = full few-shot curves for Accuracy, Macro-F1, and Macro-AUPRC (panels a–c), including comparisons with the CNN baselines. Both CNN baselines—ResNet50 and EfficientNet-B0—maintained high full-data performance but exhibited slower adaptation when trained with limited samples, showing clear sensitivity to data scarcity. In contrast, all PEFT methods improved more rapidly as k increased, with VPT showing the steepest and most consistent gains across all metrics. At k = 5, VPT already surpassed the CNN baselines and other PEFT variants, achieving higher Macro-F1 and AUPRC values despite tuning less than 1.5% of parameters. As k grew, VPT continued to widen this margin, reaching the highest accuracy and reliability at k = full. The curves converged toward the full-data endpoints reported in Table 3 (≈0.99 AUPRC and 0.99-level accuracy for VPT), while Linear, LoRA, and CNN baselines trailed by consistent margins. These results highlight that VPT adapts most effectively under label-scarce conditions, providing superior label efficiency and calibration compared to both conventional CNNs and other PEFT strategies. This behavior confirms that prompt-based tuning leverages pretrained representations more effectively than convolutional fine-tuning when annotated data are limited, an important property for practical deployment in manufacturing inspection scenarios where labeling cost is high.

### 4.3. Class-Wise Evaluation

Table 4 shows class-level accuracy patterns, including comparisons with the CNN baselines. The baseline CNNs (ResNet50 and EfficientNet-B0) performed strongly across all classes, achieving near-saturated accuracy (≥0.98 for most defect types), but their improvements plateaued quickly under full-data conditions. This confirms that while traditional convolutional models remain competitive, they require substantially larger parameter counts and do not adapt as efficiently to complex or rare defect classes.

On the CLIP-ViT-B/16 backbone, VPT achieved 0.988/0.997 on Spur and Spurious copper, outperforming Linear (0.967/0.984), LoRA (0.976/0.987), and even the CNN baselines (0.997/1.000). On DINOv2-S/14, VPT again led with 0.994/0.990 for Spur/Spurious copper, surpassing Linear (0.958/0.984), LoRA (0.944/0.974), and closely matching the CNNs (0.997–1.000).

For Short and Pinhole, all methods approached ceiling-level accuracy (~1.0), yet VPT maintained consistency across backbones (CLIP 0.997/0.997; DINO 0.983/1.000), while Linear and LoRA trailed slightly behind. Although LoRA occasionally underperformed Linear on certain classes (e.g., Mousebite on CLIP: 0.937 vs. 0.955), VPT consistently matched or exceeded both, narrowing inter-class variability and providing more balanced recognition across all defect categories.

Overall, these results show that while CNN baselines deliver excellent performance in fully supervised regimes, VPT achieves comparable or superior accuracy with over 98% fewer trainable parameters and demonstrates greater robustness to class imbalance—particularly on structurally complex defects such as Spur and Spurious copper.

### 4.4. Class-Wise Evaluation for Few-Shot Learning

**Figure 4:**
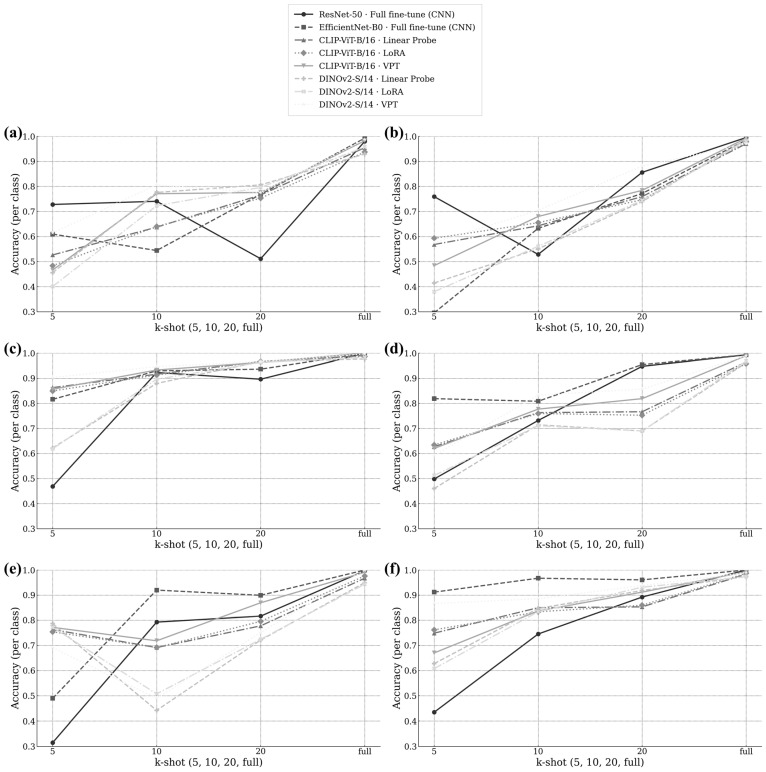
Class-wise few-shot learning curves (k = 5 to k = full) illustrating class adaptation trends for Baselines, Linear, LoRA, and VPT. (**a**) Mousebite, (**b**) Open, (**c**) Pinhole, (**d**) Short, (**e**) Spur, and (**f**) Spurious copper.

The class-wise few-shot plots in Figure 4 illustrate how each defect class responded as the number of labeled samples increased from k = 5 to k = full, including comparisons with the CNN baselines. While both CNN models (ResNet50 and EfficientNet-B0) maintained strong overall performance under full-data conditions, they showed limited adaptability when labels were scarce, with slower gains and larger fluctuations across classes at k ≤ 10.

At k = 5, the difficulty ordering mirrored the full-data results: Short and Pinhole achieved relatively high accuracy across all methods, while Spur and Spurious copper remained the most challenging. Even in this low-label regime, VPT demonstrated a clear early advantage over CNN baselines and other PEFT methods, achieving noticeably higher accuracy on the tail classes (i.e., rare or difficult-to-classify defect categories like Spur and Spurious Copper).

As k increased, VPT exhibited the steepest performance slopes on the harder classes—especially Spur and Spurious copper—closing gaps quickly and retaining this margin through k = full. In contrast, CNN baselines improved more slowly and plateaued earlier, reflecting their dependence on large scale datasets. Open and Mousebite showed steady mid-level gains with VPT, while Short and Pinhole reached near-ceiling performance by mid-k, with VPT converging faster than both CNN and other PEFT methods.

This class-wise trend reinforces the macro-level results in Figure 4: VPT not only raises the floor for difficult, low-frequency classes but also maintains top-tier accuracy on easier ones, narrowing the head–tail disparity and achieving superior label efficiency compared with both CNN and other PEFT baselines. Such behavior highlights VPT’s adaptability under label-limited conditions—a critical property for industrial PCB inspection where annotation resources are scarce.

### 4.5. Evaluation of Reliability and Calibration

Beyond averages, macro-AUPRC reflects threshold-robust reliability. At k = full, VPT achieved 0.998 on both backbones, higher than Linear (0.991/0.990) and LoRA (0.992/0.992). These results indicate better calibration between predicted confidence and correctness, supporting more stable AOI thresholding. These results are consistent with the macro-AUPRC improvements in Figure 3, approaching 1.0 for the easier classes (Short, Pinhole), matching the high AUPRC values reported.

### 4.6. Practical Implications

All three PEFT methods substantially reduced the number of trainable parameters compared to full fine-tuning:

- Linear Probe: 4614 parameters;
- LoRA: 2310 parameters;
- VPT: 5382 parameters.

Despite this efficiency, VPT achieved the highest observed values across metrics in our experiments, combining cost efficiency with high accuracy and reliability. From a deployment perspective, these results indicate that prompt tuning provides a favorable trade-off for practical PCB defect inspection, offering low training cost, rapid adaptation to new data, and broad defect coverage. Although transformer-based backbones generally exhibit slightly higher inference latency than CNN counterparts (~10–20% on average [20,21]), the PEFT variants used in this study modify fewer than 2% of parameters and maintain near-identical throughput (≈2–3 ms per patch on an RTX A6000). Therefore, the proposed VPT framework is practically deployable on industrial edge devices without measurable degradation in inspection speed.

## 5. Discussion

Traditional PCB-AOI pipelines based on CNNs or handcrafted features require large, labeled datasets and often degrade under domain shift [1,2,3]. Foundation vision models such as ViT and CLIP transfer across natural-image tasks [4,5], and self-supervised encoders like DINOv2 improve representation quality at scale [6]. However, adaptation strategies tailored to manufacturing data remain less explored. This study addresses this gap by comparing three parameter-efficient fine-tuning (PEFT) strategies—Linear, LoRA, and Visual Prompt Tuning (VPT)—on PCB defects under both full-data and few-shot regimes with class-wise analyses.

While the study’s design is a focused benchmark of three PEFT methods, this systematic comparison is a necessary contribution, as the performance of these specific adaptation strategies on manufacturing data—especially PCB defect imagery—has not been systematically assessed. Our findings provide a critical, quantitative baseline that directly addresses the gap between general-purpose vision models and the practical, data-scarce, high-reliability demands of industrial AOI, establishing a viable pathway (VPT) for real-world deployment.

With the corrected runs, VPT achieved the highest observed values across backbones and metrics. In full-data training (Table 3), VPT reached ~0.99 accuracy and ~0.998 macro-AUPRC on both CLIP-ViT-B/16 and DINOv2-S/14, whereas Linear/LoRA were ~0.966–0.972 in accuracy and ~0.990–0.992 in macro-AUPRC. Expressed in error-rate space, VPT reduced mistakes by ~64–66% relative to Linear and ~63–66% relative to LoRA (e.g., CLIP errors 2.8–2.9% → ~1.0%; DINO 3.4–3.2% → ~1.2%).

Class-wise full-data results (Table 3; Figure 3 endpoints) show that VPT increased averages and lowered inter-class spread, improving tail classes such as Spur and Spurious Copper while maintaining high scores on Short and Pinhole.

Across full-data and few-shot settings, VPT improved accuracy, macro-F1, and macro-AUPRC (Table 3 and Table 4; Figure 3 and Figure 4), in line with reports that prompt-based tuning can match or exceed heavier fine-tuning under limited labels [7]. Mechanistically, VPT injects learnable tokens at the input and propagates them through transformer layers, adjusting attention toward defect-relevant patterns without updating all pretrained weights [7]. For PCB imagery with small and irregular cues embedded in dense circuitry, these tokens may act as task-specific anchors. Two observations align with this interpretation, (i) earlier saturation on Short and Pinhole and (ii) larger gains and higher endpoints on Spur and Spurious Copper (Figure 4e–f), which are reflected in the macro-level curves (Figure 3a–c).

LoRA is parameter-efficient [8], but in our experiments, it produced lower scores than the Linear head on some classes (e.g., Mousebite under CLIP) and rarely matched VPT on tail categories (Section 4.3 and Section 4.4). This pattern suggests that PCB-specific cues benefit more from input-level, layer-pervasive conditioning (VPT) than from low-rank updates localized within layers (LoRA). Concretely, LoRA’s class-wise few-shot curves show higher variability and occasional non-monotonic trends at small k, whereas VPT shows monotonic increases that accumulate by k = full (Figure 3).

Few-shot curves (Figure 3) clarify the emergence of the full-data gap. All methods start lower at k = 5 and improve as labels increase. VPT shows the largest increases across backbones and metrics; curves converge to the full-data endpoints in Table 3. Class-wise few-shot plots (Figure 4) indicate the largest VPT gains on Spur/Spurious Copper, moderate gains on Open/Mousebite, and early saturation for Short/Pinhole, with VPT reaching the plateau earlier and maintaining higher endpoints. These trajectories explain why the VPT–baseline margin grows with k rather than saturating early.

Beyond averages, macro-AUPRC probes threshold-robust reliability. At k = full, VPT reached ~0.998 on both backbones (Table 3), higher than Linear/LoRA (~0.990–0.992). Because AUPRC is close to its maximum, small absolute gains imply improved precision–recall balance across thresholds, which is relevant for AOI where false negatives dominate cost. This view aligns with recent studies on model reliability and uncertainty in neural networks [21], supporting its relevance for industrial inspection. These findings are further consistent with studies on Vision Transformer interpretability, which link attention token interactions to prediction reliability [22,23]. Taking together, these findings highlight several practical considerations for deploying prompt-based adaptation in industrial AOI environments.

1. Label efficiency. By k = 20, VPT leads on all metrics, and the margin increases toward k = full (Figure 2 and Figure 3).
2. Training efficiency. Although the prompt head is larger than Linear/LoRA, VPT still tunes < 1.5% of backbone parameters (~5.4 k vs. 4.6 k for Linear and 2.3 k for LoRA), which is feasible on industrial GPUs.
3. Balanced performance. VPT lowers inter-class variance (Table 3), improving Spur/Spurious Copper while maintaining high scores on Short/Pinhole.
4. Backbone choice. If defects emphasize texture/edges (e.g., Spur/Spurious Copper), DINOv2-S/14 + VPT yields larger gains; for localized cues that saturate early, CLIP-ViT-B/16 + VPT is competitive (Figure 3).

While these points underscore the practicality of VPT for PCB inspection, certain aspects of our design impose limitations that future studies should address. Detection labels were reformulated as patch classification, which may omit spatial context for elongated or multi-instance defects. We evaluated two backbones on one dataset; broader testing with larger or multimodal encoders (e.g., CLIP-L/14, DINOv2 variants) would further verify generality [5,6]. Building on these findings and limitations, several directions emerge for extending this work.

- Domain shift & test-time adaptation Evaluate cross-line/factory transfer and integrate lightweight test-time adaptation (e.g., entropy minimization) to stabilize VPT under drift [24].
- Promptable detection. Extend VPT to detectors (e.g., DETR, YOLO) to retain spatial reasoning while preserving label efficiency [25,26,27].
- Calibration-aware AOI. Combine VPT with temperature scaling and risk–coverage objectives to set class-aware thresholds and enable selective routing [17,18].
- Class-conditional prompting. Allocate prompt capacity to tail classes (dynamic tokens, class-aware prompts) building on the class-wise few-shot trends [7].

As an external check on public corpora beyond DeepPCB, we plan to reproduce the protocol on HRIPCB and PCB-Defect using the same k-shot splits and metrics; the code is dataset-agnostic and requires only class-wise patch crops.

In summary, prompt-based adaptation (VPT) provides an effective accuracy–efficiency–reliability trade-off for applying foundation vision models to PCB inspection, improving tail-class performance, supporting calibration, and scaling with additional labels.

## 6. Conclusions

This study compared three parameter-efficient fine-tuning (PEFT) strategies—Linear Probe, Low-Rank Adaptation (LoRA), and Visual Prompt Tuning (VPT)—for printed circuit board (PCB) defect classification using foundation vision models CLIP-ViT-B/16 and DINOv2-S/14. Across both full-data and few-shot settings, VPT achieved the highest observed accuracy (~0.99) and macro-AUPRC (~0.998) while tuning fewer than 1.5% of backbone parameters (~5.4 k vs. 4.6 k for Linear and 2.3 k for LoRA). Error-rate analysis showed that VPT reduced misclassifications by about two-thirds, and class-wise results confirmed improved tail-class performance (Spur, Spurious Copper) while maintaining high accuracy on easier classes (Short, Pinhole). Reliability analysis further suggested improved calibration between confidence and correctness, supporting stable thresholding for automated optical inspection. Although effective, this work reformulated detection as patch-level classification and was validated on two backbones and one dataset, suggesting the need for broader testing. Future work will extend VPT to detection frameworks (e.g., DETR, YOLO), integrate calibration-aware objectives for risk-sensitive AOI, and explore domain-robust and class-conditional prompting to enhance adaptability and fairness. Overall, Visual Prompt Tuning provides an efficient and reliable pathway for adapting foundation vision models to factory-level PCB inspection, balancing accuracy, efficiency, and reliability with minimal parameter updates.

## Figures and Tables

**Table 1:** Number of defect instances per class in the training, validation, and test splits of the DeepPCB-derived dataset.

Class	Mousebite	Open	Pinhole	Short	Spur	Spurious Copper	Total
Train	1377	1353	1029	1053	1126	1037	6975
Validation	191	186	146	166	162	158	1009
Test	397	403	299	287	337	306	2029
Total	1965	1942	1474	1506	1625	1501	10,013

**Table 2:** Summary of backbone architectures.

Model	Layers	Patch Size	Embedding Dim	Pretraining Data	Pretraining Type	Key Strength
CLIP-ViT-B/16	12	16 × 16	768	400 M image–text pairs	Contrastive multimodal	Global semantic context
DINOv2-S/14	12	14 × 14	768	142 M unlabeled images	Self-distillation (SSL)	Local structure and texture

**Table 3:** Validation and test performance of Linear, LoRA, and VPT strategies on CLIP-ViT-B/16 and DINOv2-S/14 backbones under the full-data training.

Backbone	Tuning	Tunable Params	Accuracy		Macro_f1	Macro_AUPRC
			Validation	Test		
ResNet50	-	23,520,326	0.996	0.993	0.994	0.996
EfficientNet-B0	-	4,015,234	0.996	0.994	0.995	0.997
CLIP-ViT-B/16	Linear	4614	0.971	0.971	0.972	0.991
CLIP-ViT-B/16	LoRA	2310	0.970	0.969	0.971	0.992
CLIP-ViT-B/16	VPT	5382	0.991	0.990	0.991	0.998
DINOv2-S/14	Linear	4614	0.967	0.966	0.966	0.990
DINOv2-S/14	LoRA	2310	0.968	0.967	0.968	0.992
DINOv2-S/14	VPT	5382	0.995	0.988	0.988	0.998

**Table 4:** Class-wise accuracy for each defect type under the full-data training, comparing baselines, Linear, LoRA, and VPT across CLIP-ViT-B/16 and DINOv2-S/14 backbones.

Backbone	Tuning	Mousebite	Open	Pinhole	Short	Spur	Spurious Copper
ResNet50	-	0.980	0.995	1.000	0.993	0.997	1.000
EfficientNet-B0	-	0.992	0.985	1.000	0.993	1.000	1.000
CLIP-ViT-B/16	Linear	0.955	0.970	0.990	0.965	0.967	0.984
CLIP-ViT-B/16	LoRA	0.937	0.978	0.987	0.958	0.976	0.987
CLIP-ViT-B/16	VPT	0.980	0.988	0.997	0.997	0.988	0.997
DINOv2-S/14	Linear	0.929	0.983	0.980	0.965	0.958	0.984
DINOv2-S/14	LoRA	0.957	0.980	0.983	0.969	0.944	0.974
DINOv2-S/14	VPT	0.980	0.985	1.000	0.983	0.994	0.990

## Data Availability

The original data presented in the study are openly available in DeepPCB at https://github.com/tangsanli5201/DeepPCB (accessed on 26 September 2025).

## Abbreviations

ACC	Accuracy
AOI	Automated Optical Inspection
AUPRC	Area Under the Precision–Recall Curve
CNN	Convolutional Neural Network
CLIP	Contrastive Language–Image Pretraining
DINO	Self-Distillation with No Labels
ECE	Expected Calibration Error
F1	F1-Score (Harmonic Mean of Precision and Recall)
GPU	Graphics Processing Unit
LoRA	Low-Rank Adaptation
PEFT	Parameter-Efficient Fine-Tuning
SSL	Self-Supervised Learning
ViT	Vision Transformer
VPT	Visual Prompt Tuning
